# ARFID—Strategies for Dietary Management in Children

**DOI:** 10.3390/nu14091739

**Published:** 2022-04-22

**Authors:** Agnieszka Białek-Dratwa, Dorota Szymańska, Mateusz Grajek, Karolina Krupa-Kotara, Elżbieta Szczepańska, Oskar Kowalski

**Affiliations:** 1 Department of Human Nutrition, Department of Dietetics, Faculty of Health Sciences in Bytom, Medical University of Silesia in Katowice, Jordana 19, 41-808 Zabrze, Poland; eszczepanska@sum.edu.pl (E.S.); okowalski@sum.edu.pl (O.K.); 2 Poradnia Żywienia Dzieci w Bielsku Białej/Child Nutrition Clinic in Bielsko-Biała, 43-309 Bielsko Biała, Poland; poradniazywieniadzieci@gmail.com; 3 Department of Public Health, Department of Public Health Policy, Faculty of Health Sciences in Bytom, Medical University of Silesia in Katowice, Piekarska 18, 41-902 Bytom, Poland; 4 Department of Epidemiology, Faculty of Health Sciences in Bytom, Medical University of Silesia in Katowice, Piekarska 18, 41-902 Bytom, Poland; kkrupa@sum.edu.pl

**Keywords:** ARFID, feeding disorders, eating disorders, feeding and eating difficulties, child nutrition

## Abstract

Avoidant/Restrictive Food Intake Disorder (ARFID) is a relatively new disease entity in DSM-5 and ICD-11. This disorder continues to pose a diagnostic and therapeutic challenge for many professionals. This disorder can affect people of all ages. The most characteristic pattern is considered to be a lack of interest in eating or avoidance of food intake, which may result in nutritional deficiencies, weight loss or lack of expected weight gain, dependence on enteral feeding or dietary supplements, and impaired psychosocial functioning. This disorder cannot be explained by a current medical condition or co-occurring other psychiatric disorders, but if ARFID co-occurs with another disorder or illness, it necessarily requires extended diagnosis. Its treatment depends on the severity of the nutritional problem and may include hospitalization with multispecialty care (pediatrician, nutritionist, psychologist, psychiatrist, neurologist). The nutritional management strategy may include, inter alia, the use of Food Chaining, and should in the initial stage of therapy be based on products considered “safe” in the patient’s assessment. The role of the dietitian in the management of a patient with ARFID is to monitor weight and height and nutritional status and analyze the foods that should be introduced into the food chain first.

## 1. Introduction

ARFID (Avoidant/Restrictive Food Intake Disorder) is an avoidant/restrictive food intake disorder identified in the DSM V (Diagnostic and Statistical Manual of Mental Disorders) diagnostic criteria for mental disorders in 2013, which replaced feeding disorder of infancy and early childhood (FEDIC), among others [1]. In earlier DSM IV, this disorder of a somewhat similar nature was diagnosed in children up to 6 years of age [2]. ARFID does not only affect children. It can also affect adults, in whom the disorder has persisted since childhood or first appears in adulthood. Consequently, this includes some patients who did not meet the diagnostic criteria for FEDIC due to their adult age, or were classified as having an eating disorder not otherwise specified (EDNOS) [3,4]. ARFID is most often diagnosed in older children and younger adolescents; usually, pediatricians are the first to consider making the correct diagnosis. People with sensory sensitivity, in particular, may avoid eating certain foods, such as meats, vegetables, and/or fruit, due to a dislike of certain tastes, textures or smells. Other reasons for dietary restriction in ARFID may be due to lack of interest in food or low appetite [5].

## 2. Epidemiology of ARFID

The prevalence of ARFID in the pediatric population is still largely unknown, and validated screening tools are lacking. One recent study from Switzerland estimated the prevalence of ARFID among children aged 8–13 years at approximately 3.2% [6]. In specialist psychiatric and medical settings, it is estimated to range from 5–14% to 22.5% in an outpatient pediatric eating disorder treatment program. Studies have shown that it affects boys more often than girls [7,8,9,10]. The clinical characteristics of children with complex feeding difficulties are currently poorly described in the literature, making it difficult to identify and plan necessary services. Little is currently known about the rate of ARFID in adults in the general population [7,8]. Recent studies show that it affects approximately 9.2% of adult patients with eating disorders. It affects women much more often than men [11,12]. Dinkler et al. conducted a cohort screening study for ARFID. This study was conducted using a newly developed screening tool. It also attempted to estimate how many children with physical disabilities and psychosocial disorders manifested difficulties in food intake, resulting in the development of ARFID. Data were collected from 3728 children aged 4–7 years. The proportion of children with a positive ARFID screening result was 1.3%; half of these children met ARFID criteria based on psychosocial impairment alone, while the other half met diagnostic criteria for physical impairment (and in many cases additionally psychosocial impairment). Sensory sensitivity to food traits (63%) and/or lack of interest in food (51%) were the most common factors for food avoidance. ARFID-positive children were characterized by lower body weight and height, exhibited more problematic behaviors related to mealtimes and nutrient intake, were more likely to eat selectively, and were more responsive to feelings of satiety [13].

Dietary assessment should be part of the routine examination in pediatric practice, as children and adolescents are increasingly adopting restrictive dietary behaviors that carry the risk of serious nutritional deficiencies. ARFID can occur at any stage of a person’s life, but recent research has focused mainly on children and adolescents. Therefore, this systematic review focuses on children and adolescents with ARFID and the different therapies used to treat it.

The aim of this article is to review the existing knowledge on ARFID, in particular, to present the DSM-5 diagnostic criteria, the consequences of nutritional deficiency due to ARFID, and management strategies in ARFID, including dietary strategies.

We used the following methodology in our systematic review of the literature. We reviewed electronic databases including Pubmed and ScienceDirect from the last 10 years (2012–2022). The following inclusion criteria were used for the review: articles in English and Polish. The following keywords were used to search for articles: ARFID (365 articles Pubmed and 399 articles ScienceDirect), children ARFID (243 articles Pubmed and 286 articles ScienceDirect), avoidant restrictive food intake disorders (540 articles Pubmed and 630 articles ScienceDirect), and feeding disorder of infancy and early childhood (71 articles Pubmed and 5545 articles ScienceDirect). The literature review included comparative studies, cross-sectional studies, and randomized controlled trials. The last search was run on 5 January 2022.

## 3. Diagnostic Criteria

The diagnostic criteria adopted in DSM V are quite broad and should facilitate the diagnosis of an eating disorder such as ARFID. This was also the aim of the new classification of eating disorders [2]. In practice, however, there are still difficulties in diagnosing it [14]. In Table 1, we have presented the diagnostic criteria of ARFID according to DMS-V. DSM-V describes three primary symptoms of ARFID: apparent lack of interest in eating or food; food avoidance based on sensory characteristics of food; fear of aversive consequences associated with food intake which generally coincide with the Great Ormond Street criteria for feeding disorders (i.e., selective eating, emotional eating avoidance disorder, and functional dysphagia, respectively) [10].

ARFID is a disease entity, which is also included in the International Statistical Classification of Diseases and Health Problems (ICD 11) compiled by the WHO and released in 2018. ICD 11 took effect from 1 January 2022. It is listed as a disease entity under code 6B83 [15]. The diagnostic criteria in ICD 11 are compatible with the diagnostic criteria in DSM V. ICD 11 defines ARFID as a disorder characterized by avoidance or restriction of food intake, resulting in the intake of insufficient quantity or variety of food to meet energy or nutritional requirements. This may result in significant weight loss, clinically significant nutritional deficiencies, dependence on oral nutritional supplements or tube feeding, or otherwise adversely affect the physical health of a person with ARFID. It may also result in significant impairment in personal, family, social, educational, occupational, or other important functioning (e.g., avoidance or stress related to participation in social eating experiences). The pattern of eating behavior is not motivated by body weight or shape [4,16].

It is characteristic of people with ARFID to take smaller portions of food, which is usually due to the need to avoid certain foods or unpleasant interoceptive sensations. Additionally, early signaling of the sensation of satiety, lack of appetite, and anxiety during eating are significant in people with ARFID [16]. Food avoidance or restriction may be due to sensory characteristics of food (e.g., appearance, taste, smell, texture, and temperature).

ARFID is considered an eating disorder that may have a multifactorial etiology. As with other eating disorders, it is likely that both biological, medical, psychological, and environmental factors—and their interaction—contribute to the pathogenesis [17]. Several subcategories have emerged in studies published to date to define the causes of nutrient restriction [5]: selective eating since early childhood, experiencing generalized anxiety, gastrointestinal symptoms, history of vomiting or whooping, food allergy [4], insufficient intake/low interest in feeding, restricted diet due to sensory characteristics of food, and aversive/traumatic experiences [18]. Environmental and socio-cultural factors may also play an important role, e.g., feeding style including family eating style, availability of fruit and vegetables in the local environment, and exposure to healthy eating patterns and/or varied foods [17,18].

Preliminary studies conducted in pediatric clinics suggest that compared to patients with either anorexia or bulimia, cohorts of patients with ARFID tend to be younger and contain a higher proportion of the male sex, experience a longer duration of illness before presenting for treatment, and are more likely to be diagnosed with comorbidities [4,8]. In addition, an Australian interview-based study of boys and girls aged 15 years and older reported an ARFID prevalence of 0.3% in 2013 and 2014 [19]. A study of school children aged 8–13 years in Switzerland reported a prevalence of the disorder of 3.2% [6]. These data suggest that ARFID may be as common as specific eating disorders. Furthermore, studies from North America have shown that 5–12% of patients presenting to eating disorder clinics [20,21,22] and 22.5–24.6% of patients presenting to a day outpatient program for younger adolescents with eating disorders [9,23] meet criteria for ARFID.

ARFID differs from anorexia nervosa and bulimia in that there is no image disturbance. Patients with ARFID may express a desire to increase food intake and increase weight, but because of fear of foods other than those considered “safe” they cannot break through to eating other foods [1,15].

A special group of patients is children with autism spectrum disorders, in whom disorders of sensory modulation may affect the refusal to eat foods with a particular texture, taste, appearance, or smell. Such eating behaviors are common in children with ASD [24]; however, they occur with less frequency in children and adolescents without ASD [25]. In children with ASD, impaired sensory modulation may influence the inability to meet energy and nutritional needs [26]. The co-occurrence of autism may complicate the diagnosis, and therefore, requires additional clinical analysis in these patients [27,28].

Fear of the consequences of eating foods that are not considered safe by the patient contributes to avoidance/restriction of food intake. Usually, this fear concerns the situation related to vomiting after eating a given food, fear of choking, pain caused by ingested food (abdominal pain, painful defecation, reflux), and fear of allergic reaction [16,29]. Unpleasant sensations originating from the gastrointestinal tract (constipation, regurgitation) may be the reason for food refusal. The presence of unpleasant interoceptive (formerly visceral) and exteroceptive sensations (unpleasant sensations in the oral cavity resulting e.g., from hypersensitivity of the orofacial sphere after the intake of foods with specific texture or temperature) may contribute to the refusal/avoidance of food intake or early signaling of the feeling of satiety [16].

The consequence of chronic avoidance/restriction of food intake can be an inability to supply the body with sufficient nutrients and energy, which can directly result in significant weight loss or lack of expected gain in both body weight and height in children. However, Bryant-Waugh [30], in his work, suggested that ARFID can also be diagnosed in normal weight and overweight patients. This is possible because people with ARFID may consume what they consider to be safe foods, and if these are high-energy foods, this may even lead to overweight. The consumption of high-energy drinks (usually of one particular brand) in the case of adolescents or juices in the case of young children, given by carers and treated as a source of vitamins in the child’s diet, may also be a cause of overweight [30]. Correctly diagnosing ARFID in these individuals is more difficult because one of the most important criteria, significant weight loss, is not met. Usually, a person with adequate body weight or being overweight is not associated with a person who has an eating disorder, where the disorder is characterized by restricting food intake. This is particularly dangerous in the case of children. The application of body weight and height parameters on the centile grid may hide the problem the patient is struggling with. On the other hand, normal weight or being overweight does not exclude other criteria, especially the one concerning nutritional deficiencies. While the energy value of consumed meals may be sufficient, their nutritional value may indicate that they are deficient in minerals, vitamins, complete proteins, and EFAs [14,18].

## 4. Consequences of ARFID

The consequence of avoiding/restricting food intake, beyond that preferred by the patient, may be micronutrient and macronutrient deficiencies. As mentioned above, this is difficult to capture in normal weight or overweight individuals, so it is an important issue to be assessed by a physician or dietician. Health problems and abnormal development in children, resulting from a highly restrictive diet during their most intense growth and development, are dependent on which nutrients are chronically missing from the diet. Vitamin B_1_, B_2_, B_12_, C, and K deficiencies and mineral deficiencies including zinc, potassium, and iron are most commonly observed in ARFID patients. A lower intake of protein, fats, and carbohydrates is also observed, which consequently results in a lower energy value of the diet, inadequate to the patient’s energy requirements [6,31]. ARFID can lead to severe medical sequelae due to malnutrition [10]. In Table 2, we have outlined the health consequences of different dietary restrictions resulting from not providing particular food groups with the diet. Table 2 also considers how to diagnose specific deficiencies of components from the diet. 

People who exclude cereal products from their diet are at risk of low carbohydrate and fiber intake. Conversely, when dairy products are excluded from the diet, calcium may be deficient. If you exclude animal products such as meat, fish, and dairy products from your diet, you may be deficient in riboflavin, total protein, and all the amino acids needed for the body to function properly, vitamin B12, iron, selenium, and zinc. By eliminating fish from the diet, there is a deficiency of omega-3 acids and vitamin D3. If vegetables and fruit are not included in the diet, folate and vitamin C deficiencies may occur. Moreover, by eliminating both vegetable and animal fats from the diet, an adequate supply of fat-soluble vitamins such as A, D, E, and K is compromised, as well as fat, especially omega-3 fatty acids [10,24,32,33]. Many people with ARFID take vitamin supplements (e.g., multivitamins) prophylactically, so supplementation may mask the severity of malnutrition, making it difficult to assess baseline medical sequelae and their potential resolution during treatment [10,31].

Restricted food intake may result in dependence on oral nutritional supplements and, in extreme cases, enteral feeding. This may be due to the need to bypass the oral cavity and esophagus in the process of food intake, thus avoiding unpleasant sensations [1,17]. The diagnosis of significant nutritional deficiencies in children with ARFID is based on nutritional history, clinical and biological assessment (e.g., dietary intake assessment, physical examination, and laboratory tests), and the presence of clinical physical health consequences. The severity of these consequences is greater than those resulting from mental anorexia (e.g., hypothermia, bradycardia, and anemia) [34]. Restrictive behavior may induce specific deficiencies related to the nature of the excluded foods. In severe cases, especially in infants, the resulting malnutrition may even be life-threatening. The nutritional consequences of ARFID remain poorly described. Most articles have referred to low body weight or weight loss [17] Patients may report symptoms associated with acute malnutrition including fatigue, dizziness and fainting, and/or long-term malnutrition, such as abdominal pain, constipation, cold intolerance, amenorrhea, dry skin, and hair loss. On examination, signs of malnutrition may include cachexia, hypothermia, bradycardia, orthostatic tachycardia and hypotension, abdominal swelling, lanugo, and pallor [35].

Due to avoidance/restriction of food intake, family, work, and social interactions may be disrupted. Children with ARFID may avoid family gatherings, birthday parties, or school trips for fear of having to eat foods that are not acceptable to them. Fear of peer pressure causes these children to start avoiding such gatherings, gradually withdrawing from social life [1,36]. Inadequate nutritional and energy intake can also indirectly affect the psychological sphere. Insufficient growth resulting from nutrient deficiencies may be the cause of a lack of acceptance by peers. Decreased self-esteem affects the avoidance of social contact [14]. Lack of acceptance in educational institutions—where the aim is to avoid highly processed products—may cause a feeling of shame and failure in children with ARFID. Sometimes, these types of products are the only ones accepted by the child, and a lack of understanding of what the disorder is can lead to conflict.

These four diagnostic criteria help make the diagnosis. However, to make the diagnosis, it is also necessary to rule out three important aspects that are found in the DSM V and described below. ARFID cannot be explained by a lack of availability of food, e.g., for financial or housing reasons, or by a neglectful style of parenting or caring for children. Neither do religious or cultural considerations directly influence the etiology of ARFID, although, in a study by Conney et al. [29], concern for animal rights emerged as a reason for food refusal [1,29].

Both children and adults with ARFID are not concerned about their weight, and they may be unhappy with their body shape, but this is not a factor in restricted food intake. Avoidance/restriction of food intake is not related to fear of gaining weight. However, the so-called ARFID “Plus” is not excluded, which may be a consequence of the development of symptoms of anorexia nervosa [36,37]. Rare cases are also known in which ARFID has developed into anorexia [28].

A disorder such as ARFID cannot be attributed to a current illness or other psychiatric disorder if elements from the diagnostic criteria, e.g., weight loss or significant nutritional deficiencies, appear as a consequence, although this is currently debatable [1,8]. When medical factors or psychiatric disorders produce similar symptomatology, a detailed clinical analysis should be performed, which should demonstrate that the severity of the symptoms is not solely the result of the underlying disease or psychiatric disorder.

Although ARFID as a disease has existed in the DSM V diagnostic criteria for more than eight years, it is still at the beginning of its journey both diagnostically and therapeutically. Among professionals, knowledge of ARFID is still negligible. Lack of awareness results in children and adults not being properly diagnosed. Lack of proper diagnosis means a lack of proper management leading to recovery. The worsening problem adds to the frustration in families, where the child is often treated as simple non-eaters who will grow out of it. Raising awareness among both the public and professionals about the eating disorder ARFID is very important and vitally needed [14]. Just as important as the knowledge and awareness of this disorder, essential in the whole diagnostic and therapeutic process, is the understanding of what the patient with ARFID struggles with in everyday life. Understanding this disorder is essential in choosing the right management strategy, as there is still a lack of management standards for ARFID [28].

## 5. Strategies with ARFID

Eating disorders are the conditions with which patients very often first come to see a clinical dietician. Consequently, it is often the dietician/psychodietitian specializing in the treatment of eating disorders who stands at the beginning of the road leading to the correct diagnosis. The great responsibility associated with the correct management of a patient with suspected ARFID requires that the knowledge of eating disorders is sufficient to refer the patient to the appropriate specialists. A correctly made diagnosis avoids a chaotic care plan for a patient with ARFID [8].

The etiology in patients with ARFID is heterogeneous, making each case unique. Due to the high similarity of symptoms to other eating disorders or behaviors related to food intake, a quick and correct diagnosis is often difficult. While it is somewhat easier to distinguish ARFID from anorexia in adults, where, despite the complex etiology, symptoms are quite characteristic, diagnostic criteria are unambiguous, and the course of the disease known, in young children, certain behaviors such as food neophobia or picky eating are a natural stage of development. This leads to chaotic management, lack of diagnosis, and appropriate therapy, about which little is yet known [9]. In the absence of effective research-based therapy in patients with ARFID, management strategies used during therapy are needed [28,38].

In young children with ARFID who consume insufficient calories, strategies to increase dietary volume include supplementation with an oral nutritional formula, probe feeding, and behavioral interventions delivered in day treatment or hospital settings. Tube feeding is often only a temporary measure. Withdrawal is an important part of subsequent treatment. Therefore, the typical approach to weaning from tube feeding is to reduce caloric intake through tube feeding. This is to stimulate appetite for food and transition to oral feeding. Hydration and body weight should be monitored [10].

Regardless of the profession represented, the specialist should use specific and dedicated tools in making the diagnosis or when suspecting ARFID. Examples are the Nine Item Avoidant/Restrictive Food Intake disorder screen (NIAS) developed by Zickgraf et al. [39] (Table 3) and the Eating Disorders in Youth Questionnaire (EDY-Q) developed by Hilbert and van Dyck [40] (Table 4).

As feeding and eating disorders are multifactorial, it is recommended that a team of specialists work on a disorder such as ARFID. Due to the current lack of management standards, it is necessary to cooperate, among others, with a family doctor/pediatrician, gastroenterologist, psychiatrist, psychologist, dietician, neurologist, and sensory integration therapist [41]. Due to the complex nature of the disorder that is ARFID, it would be advisable for the whole team of specialists to participate in the diagnosis process, analyzing the patient’s symptoms, each in their field. The next step should be the joint development of an effective and individual therapeutic management plan. Any therapeutic management carried out in patients with ARFID should be coherent and adapted both to its age, bearing in mind the child’s development, and the severity of the course of the disease. An integral part of therapy is cooperation with the patient’s parents or caregivers and in defining the goals and expectations of each party [28].

A dietician working with pediatric patients who have feeding and eating disorders should have the necessary knowledge not to make the mistake of critically addressing the foods eaten by the patient and trying to change their eating habits. In the case of patients with ARFID, at the initial stage of therapy, it is not possible to change the products consumed for others—healthier ones. In the case of this type of patient, even if the safe products belong to highly processed foods, generally considered unhealthy, they cannot be taken away from the patient by offering a healthier alternative. In such a situation, regardless of age, the patient will choose hunger. What products are perceived as safe by ARFID patients is an important part of the therapy and should be accepted by family, the immediate environment (including nursery and school), and professionals. It is based on these products that one of the management strategies called Food Chaining will be introduced [42].

Food Chaining is a method used in feeding therapy for patients with eating disorders [42]. In the case of an ARFID patient, it is an individually tailored program, supporting pharmacological treatment as well as psychotherapy, aimed at broadening the range of foods by emphasizing similar characteristics between a safe product and one that we want (or the patient wants) to introduce into the patient’s diet. This is done in a variety of ways that depend, among other things, on the patient’s age and health status. Food Chaining will not always be the beginning of the therapy. Patients with a high fear of eating other than safe products require some kind of preparation, to get used to the new situation [42].

In the case of young children, the therapy involves familiarizing them with new foods through play, e.g., drawing, coloring, and playing with toys that look like food. This kind of play reduces anxiety, helps to reassure the child, and prepares them for exposure to the product to be eaten. It may take up to several months before the child is ready for exposure to a real food product. Note that children with ARFID are more resistant to exposure techniques than children with food selectivity [16].

To reduce anxiety in ARFID patients, working with Food Chaining should in the initial stage be based on safe products, but differently than before. If a patient’s diet consists of, for example, toast with cheese, waffles, grilled chicken breast, vegetable soup with white rice, and crinkle fries, then in the first stage, we work with the patient only on these products, making very slight modifications with the patient’s consent. A sample Food Chaining might then look as follows: toast with a small amount of butter and cheese, grilled turkey breast, waffles using a different flour, e.g., whole grain, vegetable soup with brown rice, straight fries. Of course, changes are made one at a time and gradually, taking into account the patient’s tolerance to the changes made. For an ARFID patient, this will be a big change, but still within safe foods, which minimizes feelings of anxiety. Subsequently, the range of foods can be gradually expanded depending on the patient’s condition, motivation, and behavioral problems. However, this must be gradual and accepted by them to the extent that he is willing to cooperate. It is very helpful to have a routine to follow, as it contributes to a sense of security. You cannot surprise the patient by hiding other products that are safe for him, which will be identified by them anyway. People with ARFID accept a particular taste, smell, form of serving, temperature, or consistency of a given food, usually always prepared by the same person. At the same time, they accept only a particular type or brand of product, and even a slight change can contribute to its rejection [16,42].

All progress and outcomes should be carefully monitored. The role of the dietitian is to monitor weight and height and nutritional status and to analyze the foods that should be introduced into the food chain first to minimize malnutrition, bearing in mind their similarity to safe foods.

Therapeutic management of the youngest patients with ARFID is as difficult as working with older children. Few scientific studies have been conducted with the youngest group of patients diagnosed with feeding and eating disorders. According to Chatoor [43], in children over 1 year to 3.5 years of age, an important part of therapy is to understand the child’s temperament and associated hyperactive behavior. Lowering the child’s emotions before or during meals, i.e., eating in an appropriate atmosphere, can help to introduce other food components into the child’s diet [43].

Another innovative management strategy is that of Zucker [16], who in her research focused on slightly older children (4 to 10 years old), paying attention to interoceptive sensations, proposing a method based on sensations and internal body sensations “The Feeling and Body Investigators” (FBI) [16]. Interoceptive exposure therapies developed for anxiety and gastrointestinal disorders may provide an appropriate framework to address problems related to avoidance/restriction of food intake resulting from unpleasant body sensations. In FBI therapy, both interoceptive and exteroceptive sensations and feelings should be perceived by the child as something interesting rather than something that causes fear. Therefore, in therapy, the focus should be on experiencing and exploring unpleasant body sensations, and instead of interrupting them, the child should be taught acceptance through play. An important point is that interoceptive sensations are informational and send signals to the brain about hunger, satiety, fatigue, and emotions, among others. Teaching self-awareness by mapping interoceptive states can reduce feelings of anxiety and increase acceptance of these sensations. Playing FBI agents with “sensory superpowers” allows the exploration of body sensations. Cartoon characters can be used to represent different bodily sensations identified in a body map. For example, hunger, identified on a body map in the abdominal area, can be communicated by a mascot such as Chase from the Dog Patrol sitting on a child’s tummy and announcing through a megaphone that they are hungry. Chase informs them that they should go to the kitchen and eat a meal to appease their hunger. A worksheet for playing FBI detective is available in the author’s original publication [16]. The proposed method does not apply to all ARFID patients due to the different nature of the symptoms. However, in situations involving hypersensitive interoceptive and exteroceptive sensations, this method can be of great benefit [16].

An extremely important part of the management of patients diagnosed with ARFID is not to recommend starvation as part of the therapy. This practice is not effective and, in the case of an eating disorder such as ARFID, can be dangerous.

Methods of working with the patient based on the above strategies should be supported by psychotherapeutic therapies. An example is an application in the case of ARFID of the “Family-Based Treatment” (FBT), which was described in detail by a team of researchers, Rosania and Lock [44], from Stanford University, USA. FBT is a method widely used for specific eating disorders, such as anorexia or bulimia. The authors of the publication suggest that this therapeutic model can also be used to treat ARFID. FBT in the treatment of specific eating disorders mobilizes families to help the child overcome the disorder in three stages.

Stage 1 focuses on restoring normal weight by having parents take control of their child’s eating. In the first session, the therapist emphasizes the seriousness of the disorder to increase the parents’ sense of responsibility. The therapist takes an agnostic approach, emphasizing that the cause of the disorder is unknown, to reduce parental guilt and redirect their attention from focusing on potential causes. The therapist externalizes the eating disorder, presenting it as separate from the child and not under their control. The second session includes a shared family meal and allows the therapist to assess family dynamics that may have an impact on weight restoration.

Stage 2 occurs when the child is eating without much resistance, weight gain is steady and parents feel empowered to manage symptoms. It focuses on helping parents to restore their child’s control of overeating in a way that is age-appropriate and consistent with their needs.

Stage 3 occurs when the child’s weight has returned to normal, the behaviors associated with the eating disorder have subsided, and the child is self-managing their eating and physical activity. The focus is on addressing adolescent developmental issues that have been disrupted by the disorder.

The FBT-ARFID therapeutic model differs somewhat from the classical one. Treatment goals focus on eating behaviors (e.g., increasing variety) rather than on restoring normal weight. The child may provide more helpful information during treatment and be more sensitive to rewards than, for example, children with anorexia. Finally, phase 2 and phase 3 are different: phase 2 occurs when the child can consistently try new foods; phase 3 often does not occur because ARFID often occurs before puberty.

It is worth noting that ARFID is quite heterogeneous, and the use of FBT-ARFID in patients with aversive food anxiety and/or low appetite may require adaptation. Although basic FBT interventions including parental reinforcement appear appropriate for any form of ARFID, modifications to more specific interventions (e.g., weight gain, increased dietary flexibility, family meals) may be warranted. Further guidance on treatment in different forms of ARFID can be found in the case reports of Lock and colleagues [45].

The three-dimensional neurobiological model of ARFID can be used in nutritional and therapeutic management. Data support the three-dimensional model of ARFID, in that ARFID varies in severity and that nearly half of the people with ARFID who present for psychological treatment exhibit eating difficulties across multiple ARFID ranges [10,46,47].

People with ARFID sensory sensitivity often describe non-preferred foods as intensely negative in taste. The traditional clinical understanding of this presentation is that people with sensory sensitivity simply have no experience with non-preferred foods and will prefer them on repeated exposure. Indeed, there is evidence that adults who identify as picky rate both bitter and sweet tastes as significantly more intense than individuals who do not identify as picky [10,47].

People with a lack of interest in food or ARFID often describe not feeling hungry at mealtimes, forgetting to eat, and/or feeling full faster than others. It has been hypothesized that lack of interest in ARFID may be related to differences in the activation of brain centers regulating appetite. In this group, few individuals will develop ARFID, suggesting this subgroup may have entered the traumatic eating experience with a pre-existing sensitivity that gave rise to a phobic response. Psychophysiological reactivity to fearful stimuli distinguishes phobic-type anxiety disorders (i.e., social and specific phobias) from other anxiety disorders. Given a three-dimensional neurobiological model, those treating patients with ARFID can tailor treatment plans so that therapy targets the basis of biological abnormalities in sensory sensitivity, low appetite, and food concerns [10,47].

The best documented and effective form of help for patients with ARFID is cognitive-behavioral therapy (CBT) [46]. Given that anxiety is prevalent in some patients, the use of CBT seems to be an option to work through the difficulties present, especially in children. CBT-AR used on an outpatient basis is recommended for patients aged 10 years and older, medically stable, and not requiring tube feeding. CBT-AR treatment in ARFID includes 20 to 30 sessions, with severely underweight patients requiring more sessions to introduce work on weight gain. Therapeutic management is divided into four stages.

Stage I consists of psychoeducation of the patient and their carers related to ARCID and the CBT-AR therapy used. In underweight patients, an intervention is undertaken to increase daily intake by 500 kilocalories to achieve a weight gain of 0.5–1.0 kg per week. Collaboration with a dietitian is necessary at this stage. In patients with normal and stable body weight, a set of preferred foods is undertaken and initial changes are planned.

Stage II consists of continuing psycho-education related to nutritional deficiencies and health consequences resulting from ARFID. An analysis of the range of foods and dishes acceptable to the patient takes place and further changes are planned.

In Stage III, the patient, together with the therapist, creates a list of foods and situations that cause the medication to prioritize them from least to most uncomfortable. Exposures to the food product/food take place in the office and are continued at home. For patients who are not interested in food, interoceptive exposures are used to familiarize the patient with body sensations such as those associated with a filling stomach.

Stage IV involves assessing progress and developing a relapse prevention plan [46,47].

Regardless of the method used (Food Chaining, FBI, CBT, CBT-AR, FBT-ARFID), a very important therapeutic factor in therapy is the trust and cooperation of parents in the therapeutic process [48]. It is important to remember that parental pressure associated with the expectation that their child will eat everything negatively affects their eating behavior. This in turn correlates with the frequency of picky eating and lower average body weight. This can contribute both to dissociation of eating and hunger/satiety symptoms and poor eating habits for external reasons such as emotions [49]. Parents need to be aware that the pressure they exert is often more related to their anxiety than to the nutritional risks to their children. Informing parents of the detrimental effects of exerting pressure and checking that children are eating a balanced diet should be part of routine clinical practice in pediatrics [5,24].

**Figure 1:**
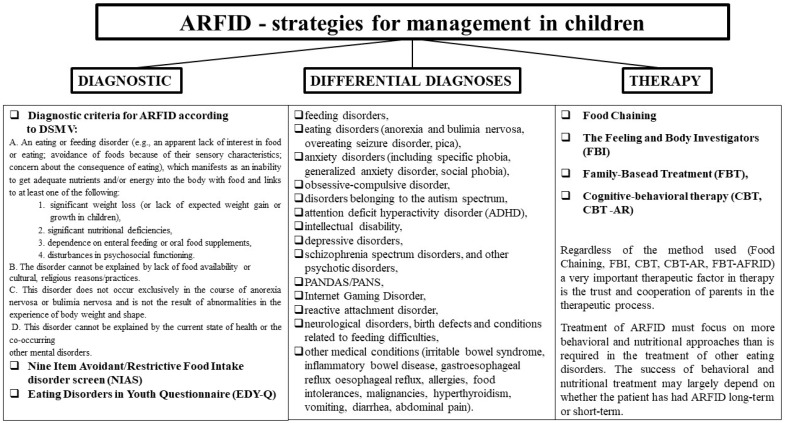
ARFID—strategies for therapeutic management in children.

Treatment of ARFID must focus on more behavioral and nutritional approaches than is required in the treatment of other eating disorders. There are several studies documenting the clinical outcomes of ARFID. A review of medical records for emergency hospital admissions found that adolescents with ARFID required longer hospital stays and were more likely to require enteral feeding for stabilization compared with those with AN [50]. In the Formann study, adolescents with ARFID presenting to adolescent health services were observed for a shorter period and were less likely to reach normal weight compared with those with AN or atypical AN [20]. There are many studies on the treatment of eating disorders in children including young children, in particular behavioral interventions to increase dietary volume and variety. However, no randomized controlled trials have evaluated the effectiveness of any type of ARFID treatment in adolescents or adults. Single case reports have highlighted the potential effectiveness of cognitive behavioral therapy and family therapy for adolescents with ARFID, though larger-scale studies are lacking [10]. The success of behavioral and nutritional treatment may largely depend on whether the patient has had ARFID long term or short term. Long-term patients require more intensive treatment over a longer period. The main aim of their treatment may be to minimize some of the symptoms instead of eliminating the disorder. The next steps in therapy must be individually tailored to the patient’s needs, realistic and achievable, and should include mitigation of nutritional, physical, and emotional risks while helping the patient manage anxiety and expand eating [5,30]. In summarizing the management strategy for ARFID we have included the process of diagnosis, diagnostic differentiation and therapy (Figure 1).

## 6. Conclusions

Patients with ARFID have different treatment and management needs, which depend on the condition, the patient’s age, and the specifics of the accompanying symptoms. The patient must be managed by a team of professionals who will take into account not only physical health and nutritional improvement, but also pay attention to psychological factors. Understanding the patient with an eating disorder such as ARFID seems to be an extremely important aspect of the whole therapeutic process. As part of pediatric follow-up, dietary screening should be systematically carried out to prevent both nutritional and psychological effects of ARFID.

## Figures and Tables

**Table 1:** Diagnostic criteria for ARFID according to DSM V [1].

A. An eating or feeding disorder (e.g., an apparent lack of interest in food or eating; avoidance of foods because of their sensory characteristics; concern about the consequence of eating), which manifests as an inability to get adequate nutrients and/or energy into the body with food and links to at least one of the following: 1. Significant weight loss (or lack of expected weight gain or growth in children); 2. Significant nutritional deficiencies; 3. Dependence on enteral feeding or oral food supplements; 4. Disturbances in psychosocial functioning.
B. The disorder cannot be explained by lack of food availability or cultural and religious reasons/practices.
C. This disorder does not occur exclusively in the course of anorexia nervosa or bulimia nervosa and is not the result of abnormalities in the experience of body weight and shape.
D. This disorder cannot be explained by the current state of health or other co-occurring mental disorders.

ARFID: Avoidant/Restrictive Food Intake Disorder. DSM V: Diagnostic and Statistical Manual of Mental Disorders 5th ed.

**Table 2:** Signs and symptoms of specific vitamin-mineral deficiencies due to dietary restrictions [10,24,32,33].

Type of Food Avoided	Nutrient Deficiency	Basic Parameter	Health Consequences of Deficiency
**Cereal products**	carbohydrates	body weight and height	hypotrophy
	fiber	e.g., screening for cancer, atherosclerosis, cholelithiasis	Atherosclerosis, gallstones, diverticulosis, and colorectal cancer, breast cancer in women.
**Milk and milk products**	calcium	PTH, alkaline phosphatases	rickets, hypocalciuria, reduced bone mineral density, osteopenia, bone weakness or fractures, and osteoporosis.
**Animal products and dairy products**	Riboflavin/Vitamin B2	the serum concentration of vit. B2	Low energy levels, poor growth, dry skin/skin problems, hair loss, dry cracked lips or cracks at the corners of the mouth, magenta tongue swelling, itchy and/or red eyes, sore throat, loss of lean body mass, anemia, and cataracts
	total protein	Plasma protein, albumin, prealbumin	malnutrition, edema
	vitamin B12 Cobalamin	plasma cobalamin	Hyperhomocysteinemia, megaloblastic or macrocytic anemia, low energy, weakness, numbness or tingling in hands or feet, difficulty walking or instability, constipation, anorexia, confusion, poor memory, mood changes, psychosis, and mouth/tongue discomfort
	Iron	Plasma ferritin, the plasma iron	Microcytic anemia, pallor, weakness, fatigue or drowsiness, irritability, poor concentration, learning, cognitive difficulties, mood changes, reduced exercise endurance, headaches, temperature intolerance, weakened immune system, and reduced appetite due to mucosal changes (disappearance of tongue papillae with taste buds, reduced saliva production)
	Selenium	Selenium in plasma	Oxidative stress
	Zinc	Plasma zinc	Oxidative stress, poor growth, and development, anorexia, weakened immune system, impaired night vision, taste and smell changes, hair loss, diarrhea, and poor wound healing
**Fish**	omega-3 acids	omega-3 acids in plasma	central nervous system disorders and cardiovascular disorders
	vitamin D3	plasma vitamin D3	rickets, osteomalacia, and osteopenia
**Vegetables and fruits**	folates	plasma folate	Hyperhomocysteinemia, megaloblastic or macrocytic anemia, persistent fatigue, pallor, palpitations, dyspnoea, headache, mouth ulcers, increased risk of birth defects, poor concentration, increased irritability, and weight loss
	Vitamin C	Vitamin C in plasma	Microcytic anemia, scurvy, petechiae, easy bruising, bleeding, and swollen gums, anorexia, anemia, malaise, muscle, joint pains, corkscrew, perianal hemorrhage, wound healing disorders, hyperkeratosis, weakness, and mood disorders
**Animal and vegetable fats**	Vitamin E	Vitamin E/lipids	Oxidative stress
	Vitamin A	Plasma vitamin A	Hemeralopia, poor night vision/night blindness, weakened immune system, hyperkeratosis, and impaired wound healing
	Vitamin K	Plasma vitamin K	Bruising and easy bleeding and prolonged prothrombin time
	Fat	observation	Weight loss and absence of menstruation

PTH: Parathyroid hormone (PTH).

**Table 3:** NIAS questionnaire [39]. Nine Item Avoidant/Restrictive Food Intake disorder screen (NIAS)—Child.

		STRONGLY DISAGREE	DISAGREE	SLIGHTLY DISAGREE	SLIGHTLY AGREE	AGREE	STRONGLY AGREE
1	I am a picky eater	□	□	□	□	□	□
2	I dislike most of the foods that other people eat	□	□	□	□	□	□
3	The lisf of foods that I like and will eat is shorter that the list of foods I won’t eat	□	□	□	□	□	□
4	I am not very interester in eatingl I seem to have a smaller appetite than other people	□	□	□	□	□	□
5	I have to push myself to eat regular meals throughout the day, or to eat a large enough amount of food at meals	□	□	□	□	□	□
6	Even when I am eating a food I really like, it is hard for me to eat a large enough volume at meals	□	□	□	□	□	□
7	I avoid of put off eating because I am afraid of GI discomfort, choking, or vomiting	□	□	□	□	□	□
8	I restrict myself to certain food because I am afraid that other foods will cause GI discomfort, choking, or vomiting	□	□	□	□	□	□
9	I eat small portion because I am afraid that other foods will cause GI discomfort, choking, or vomiting	□	□	□	□	□	□

**Table 4:** EDY-Q questionnaire [40].

Please Read Through the Following Statements and Check the Box that Describes You Best.		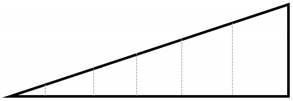					
		Never True					Always True
1	If I was allowed to, I would not eat.	□	□	□	□	□	□
2	Food/eating does not interest me.	□	□	□	□	□	□
3	I do not eat when I’m sad, worried, or anxious.	□	□	□	□	□	□
4	Other people think that I weigh too little.	□	□	□	□	□	□
5	I would like to weigh more.	□	□	□	□	□	□
6	I feel fat, even if other people do not agree with me.	□	□	□	□	□	□
7	As long as I do not look too fat or weigh too much, everything else does not matter.	□	□	□	□	□	□
8	I am a picky eater.	□	□	□	□	□	□
9	I do not like to try new food.	□	□	□	□	□	□
10	I am afraid of choking or vomiting while eating.	□	□	□	□	□	□
11	I am afraid of swallowing food.	□	□	□	□	□	□
12	I do not like to try food with a specific smell, taste, appearance, or a certain consistency (e.g., crispy or soft).	□	□	□	□	□	□
13	I like to eat things that are not meant for eating (e.g., sand).	□	□	□	□	□	□
14	I regurgitate food that I have already swallowed.	□	□	□	□	□	□

## Data Availability

Not applicable.
